# Enhancing acute inflammatory and sepsis treatment: superiority of membrane receptor blockade

**DOI:** 10.3389/fimmu.2024.1424768

**Published:** 2024-07-16

**Authors:** Seok-Jun Mun, Euni Cho, Hyo Keun Kim, Woo Jin Gil, Chul-Su Yang

**Affiliations:** ^1^ Department of Bionano Engineering, Hanyang University, Seoul, Republic of Korea; ^2^ Center for Bionano Intelligence Education and Research, Hanyang University, Ansan, Republic of Korea; ^3^ Department of Molecular and Life Science, Hanyang University, Ansan, Republic of Korea; ^4^ Department of Medicinal and Life Science, Hanyang University, Ansan, Republic of Korea

**Keywords:** sepsis, membrane receptor blockade, damage-associated molecular patterns, pathogen-associated molecular patterns, inflammation

## Abstract

Conditions such as acute pancreatitis, ulcerative colitis, delayed graft function and infections caused by a variety of microorganisms, including gram-positive and gram-negative organisms, increase the risk of sepsis and therefore mortality. Immune dysfunction is a characterization of sepsis, so timely and effective treatment strategies are needed. The conventional approaches, such as antibiotic-based treatments, face challenges such as antibiotic resistance, and cytokine-based treatments have shown limited efficacy. To address these limitations, a novel approach focusing on membrane receptors, the initiators of the inflammatory cascade, is proposed. Membrane receptors such as Toll-like receptors, interleukin-1 receptor, endothelial protein C receptor, μ-opioid receptor, triggering receptor expressed on myeloid cells 1, and G-protein coupled receptors play pivotal roles in the inflammatory response, offering opportunities for rapid regulation. Various membrane receptor blockade strategies have demonstrated efficacy in both preclinical and clinical studies. These membrane receptor blockades act as early stage inflammation modulators, providing faster responses compared to conventional therapies. Importantly, these blockers exhibit immunomodulatory capabilities without inducing complete immunosuppression. Finally, this review underscores the critical need for early intervention in acute inflammatory and infectious diseases, particularly those posing a risk of progressing to sepsis. And, exploring membrane receptor blockade as an adjunctive treatment for acute inflammatory and infectious diseases presents a promising avenue. These novel approaches, when combined with antibiotics, have the potential to enhance patient outcomes, particularly in conditions prone to sepsis, while minimizing risks associated with antibiotic resistance and immune suppression.

## Graphical Abstract

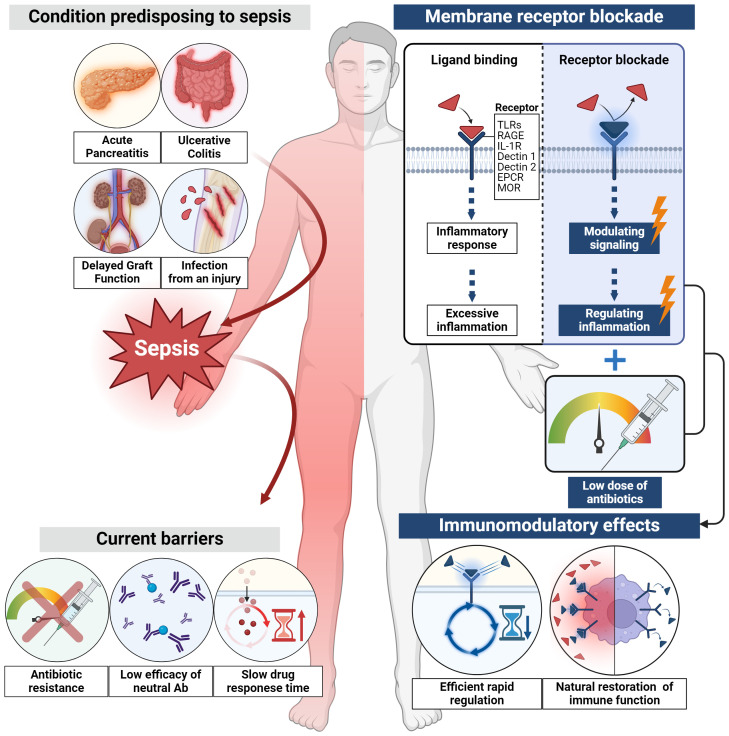

## 1 Introduction

The intricate interplay between acute inflammatory diseases, infectious diseases, and the development of sepsis poses a serious challenge in modern healthcare (1). The timely intervention in these diseases after their inception is of paramount importance (2–4). Without initial treatment, these diseases progress and a substantial number of patients develop sepsis, which is a life-threatening condition due to a dysregulated host response to infection (5). Sepsis has a high mortality rate (6). Sepsis affects nearly 49 million people globally each year, resulting in approximately 11 million deaths. These figures highlight sepsis as one of the major causes of mortality worldwide, accounting for about 20% of all deaths (7). The economic impact is substantial, with global healthcare systems incurring billions of dollars annually due to long hospital stays and intensive medical interventions (8). Particularly in regions with limited healthcare infrastructure, the burden of sepsis is high, and the prevalence of antibiotic-resistant infections further exacerbates these challenges (9). Antimicrobial resistance (AMR) significantly impedes effective treatment options, extending the duration of illness and increasing both mortality rates and healthcare costs (10). Understanding the biological mechanisms that lead from initial infection to sepsis is crucial. This involves delving into the molecular intricacies that govern the immune response during the early stages of these diseases.

Intricate signaling pathways are involved in the early stages of these diseases. Intracellular signaling cascades are initiated by the recognition of endogenous damage-associated molecular patterns (DAMPs) or exogenous pathogen-associated molecular patterns (PAMPs) by pattern recognition receptors (PRRs) (11, 12). This cascade activates immune responses involving cytokines, alarmins, and other mediators, aimed at eliminating pathogens and maintaining cellular homeostasis (13, 14). However, within the context of inflammatory diseases, certain signaling pathways are dysregulated, leading to the overproduction of proinflammatory cytokines and alarmins (15). The resulting excessive systemic inflammation and immune suppression contribute to disease severity (16). Given the complexities of these pathways, it becomes evident why traditional treatment strategies often fall short. This segues into a discussion of how conventional treatments typically approach these challenges, and why there’s a pressing need for innovative therapeutic strategies.

The conventional treatments for sepsis, which includes acute inflammatory and infectious diseases, often involve the use of antibiotics such as cefepime, piperacillin-tazobactam, and ceftobiprole as the primary intervention (17, 18). These antibiotics work by targeting and killing the bacteria responsible for the infection, thus reducing the bacterial load and aiding in infection control. Additionally, standard sepsis treatments include supportive measures like fluid resuscitation, vasopressors, mechanical ventilation, and renal replacement therapy to stabilize patients and maintain vital organ function, but these do not directly address the underlying infection (19, 20). These supportive measures are essential for maintaining patient stability and managing the immediate life-threatening symptoms of sepsis. However, they do not directly target the underlying infection or the immune dysregulation associated with sepsis. In addition to these challenges, the efficiency of antibiotic treatments may be impaired due to the emergence of antibiotic resistance and delayed drug response. For instance, methicillin-resistant Staphylococcus aureus (MRSA) and carbapenem-resistant Enterobacteriaceae (CRE) are examples of antibiotic-resistant bacteria that complicate sepsis treatment, leading to higher mortality rates (8, 21). These resistant strains are not effectively killed by standard antibiotics, necessitating the use of alternative or combination therapies, which may not always be readily available or as effective. Moreover, delays in administering appropriate antibiotics, often due to the time required for microbial culture and sensitivity testing, can result in worsening patient outcomes. Early and appropriate antibiotic administration is critical for improving sepsis survival rates; however, the need for precise identification of the causative pathogen can lead to significant delays in treatment initiation (22–24). To address these issues, recent approaches have focused on the coadministration of antibiotics with other therapeutic agents that can modulate the immune response. Inflammatory regulators are a representative example of such combination therapies. These agents target various components of the inflammatory signaling pathway, including membrane receptors and intermediate substances (25–28). This evolution in treatment underscores the growing importance of targeting specific components of the inflammatory cascade, which leads us to examine innovative methods for inflammation regulation that address broader issues such as sepsis and antibiotic resistance.

Among the new methods for regulating inflammation, membrane receptor blockade can be used to address the issues related to sepsis, antibiotic resistance, and the use of conventional treatments (29). Therapeutic candidates targeting membrane receptors show the ability to rapidly control inflammation (30–32), so could be effectively applied for the treatment of acute inflammatory diseases, infectious diseases, and sepsis, for which the initial treatment is crucial. Membrane-receptor-targeting substances have immunomodulatory abilities to restore immune function rather than just inducing complete immunosuppression (33, 34). It is important to distinguish between immunosuppressants and immunomodulators. Immunosuppressants are characterized by low therapeutic indexes, meaning there are close windows between therapeutic ranges and toxic zones. They also exhibit significant intra- and interindividual drug kinetic variations. These shortcomings are mitigated by correct drug doses, which are calculated based on ideal body weight (a calculated weight considered optimal for health) or lighter body weight (used to avoid overdosing in underweight patients). Additionally, end-organ toxicity testing and, in some cases, close monitoring of plasma drug levels (parent or metabolite peak and depth levels) are used. In contrast, immunomodulators have a broader therapeutic index, a higher safety margin, more predictable drug behavior properties and fewer intra-individual variability. In addition, although immunosuppressants typically affect host immune reactions globally, immunomodulators can act selectively on specific parts of the immune system and therefore reduce the risk of complications related to immune failure (35). In this context, the drugs investigated in the referenced papers did not induce cytotoxicity or cell death.

This review presents new therapeutic approaches for acute inflammatory and infectious diseases that increase the risk of sepsis, as well as complementary methods to enhance current sepsis treatments.

## 2 Methodology

For this review, we utilized PubMed and ClinicalTrials.gov to search the literature using specific terms, including ‘sepsis,’ ‘membrane receptor blockade,’ ‘damage-associated molecular patterns,’ ‘pathogen-associated molecular patterns,’ and ‘inflammation.’ Our focus was on English-language articles that present original research, prioritizing significant and relevant studies in the domain. Although our search was not restricted by publication date, we made a concerted effort to cite the most recent studies, generally avoiding references older than ten years.

## 3 Acute inflammatory and infectious diseases, and their correlation with sepsis

Acute pancreatitis (AP) often progresses to severe acute pancreatitis (SAP), where systemic inflammation frequently leads to multiple organ dysfunction syndrome (MODS) and, in some instances, sepsis, significantly increasing mortality in about 20% of these cases (36, 37). Moreover, AP results in the breakdown of bacterial translocation and intestinal integrity, and an increased risk of infection (38). Ulcerative colitis (UC) is a chronic inflammatory condition that can lead to acute kidney injury, which is associated with septic shock (39). In addition, delayed graft function (DGF), which occurs in one in five patients receiving organ transplant, provides a favorable environment for sepsis because repeated biopsies of the transplanted organs are required (40). Patients with acute inflammatory and infectious diseases and those who have undergone DGF frequently develop sepsis, a systemic inflammatory disease with a remarkably high mortality rate (41). Furthermore, infections caused by injury can lead to sepsis. Sepsis can occur due to other infections, such as viruses and fungi, but most infections are caused by bacteria. Bacteria are classified into Gram-negative and Gram-positive types and are both capable of causing sepsis; however, they have distinct differences. The most commonly isolated bacteria in sepsis are *Staphylococcus aureus* (*S. aureus*)*, Streptococcus pyogenes* (*S. pyogenes*)*, Klebsiella species* (*Klebsiella* spp.)*, Escherichia coli* (*E. coli*)*, and Pseudomonas aeruginosa* (*P. aeruginosa*). To cause disease, pathogens must use a number of factors called virulence factors to protect themselves from host innate immune system, to cross mucous membrane barriers, to spread, and to replicate to distant organs (42, 43). To understand the factors causing bacterial pathogenicity, it is crucial to study how pathogenic agents escape host immune systems, cross mucous membrane barriers, spread, and replicate in distant organs. For example, *S. aureus* and *S. pyogenes* infect primarily the skin and lung and activate toll-like receptor (TLR) 2. Some alleles of the TLR2 pathway are associated with an increase in sensitivity and severity to sepsis caused by large Gram-positive pathogens such as *S. aureus* (44–47). *Klebsiella* spp. infects a variety of sites including the lungs, urinary tracts, blood vessels, wounds, surgery, and the brain. TLR2 recognizes the OmpA of *Klebsiella pneumoniae* (*K. pneumoniae*) and activates the NF-κB signal, while TLR4 recognizes the LPS and K1-CPS of *K. pneumoniae* (48–50). *E. coli*, commonly linked to intestinal diseases, also significantly contributes to systemic infections like sepsis. Additionally, pathogenic *E. coli*, especially strains like uropathogenic *E. coli* (UPEC), can cause severe conditions such as bacteremia and sepsis. These extraintestinal infections caused by pathogenic *E. coli* are the leading cause of sepsis in healthcare environments and communities (51–53). *P. aeruginosa* is a common pathogen found in severe burn injuries and is associated with various nosocomial infections such as pneumonia, surgical wounds, urinary tract infections, and bacterial infections. Signals induced by TLR4 and flagellin mediate the acute inflammatory response of pseudomonas, while TLR2 plays a counter-regulatory role. The myeloid differentiation primary response 88 (MyD88)-dependent pathways, as well as the pathways downstream of TLR2, TLR4 and TLR5, are necessary for lung defense against *P. aeruginosa* (54, 55). Sepsis is an inflammatory response in the host to serious infections that pose a threat to life and accompany organ dysfunctions (56) and initiates a complex interaction of host pro-inflammatory and anti-inflammatory processes. Sepsis involves dynamic interaction between the host immune system and pathogens (57), the result of which depends on a delicate balance between anti-inflammatory and pro-inflammatory pathways. Septic shocks are a subgroup of sepsis with profound circulatory, cellular and metabolic abnormalities that are associated with a higher mortality risk than sepsis alone. In the current definition of sepsis, the term “abnormal regulation and host response” is not explicitly defined, but is conceptualized as a maladaptive response within the immune and non-immune systems that leads to organ failure and death (58). In the sepsis context, adherence to an initial treatment response timeframe is crucial for controlling mortality rates (59). As such, there is an increasing need for a novel approach that includes the use of inflammatory regulators as adjunctive therapy with antibiotics, not only to manage the excessive inflammation caused by sepsis but also to prevent septic shock and organ dysfunction, thereby effectively controlling and preventing the progression of sepsis within the required timeframe.

## 4 Signaling pathways and limitations of acute inflammatory and infectious disease treatment

**Figure 1:**
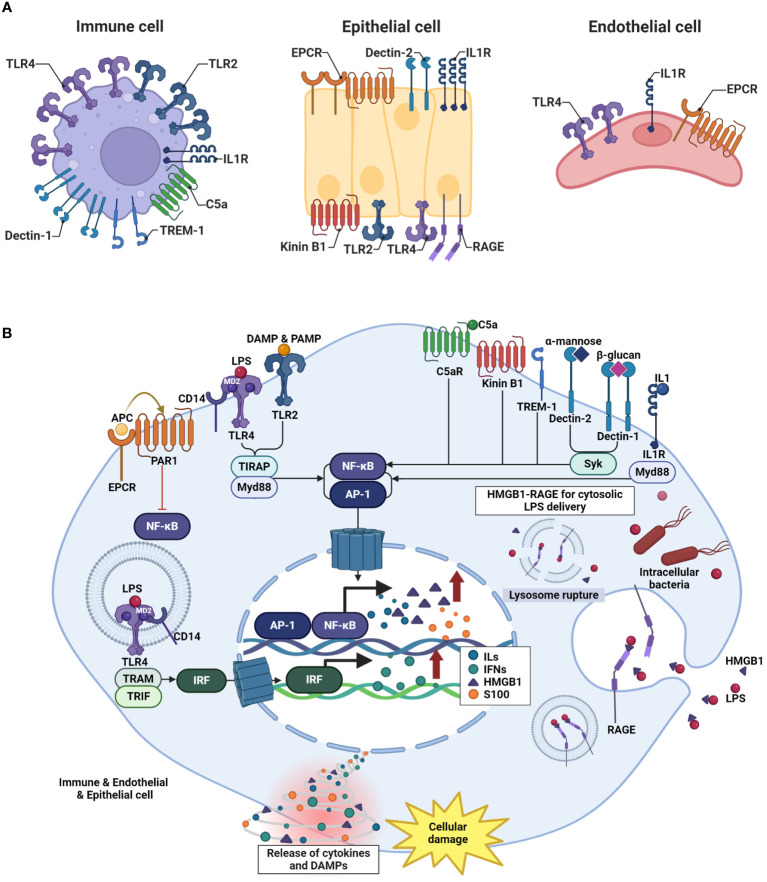
Mechanism of inflammatory and infectious diseases develop. **(A)** Variations in pattern recognition receptor (PRR) expression are cell-specific. The receptors named for each cell type reflect their high level of expression, as determined by the Human Protein Atlas data. **(B)** In the early stages of inflammatory and infectious diseases, the host recognizes DAMPs or PAMPs. DAMPs and PAMPs directly bind to membrane receptors such as TLRs, IL-1R, C-type lectin receptors, RAGE, TREM-1, and GPCR. This interaction triggers the activation of key signaling pathways, including IRF, responsible for IFN production, NF-κB signaling pathway. Signaling pathways are significantly upregulated, resulting in the robust release of cytokines such as TNF-α, IL-1, and IL-6, as well as DAMPs like HMGB1 and S100 family proteins.

When inflammatory and infectious diseases develop, a cascade of events is set in motion, including the release of DAMPs and PAMPs, leading to cellular damage (**Figure 1**). At the onset of these diseases, the host recognizes endogenous DAMPs or PAMPs, which interact with membrane proteins such as a series of PRRs located on the cell membrane or in the intracellular space (60, 61). Intracellular signaling pathways are activated, culminating in the production of diverse end-products with the potential to serve as DAMPs. DAMPs and PAMPs interact and directly bind to membrane receptors such as TLRs, interleukin-1 receptor (IL-1R), C-type lectin receptors (dectin 1 and dectin 2), and receptor for advanced glycation end products (RAGE) (62–64). As a result of these interactions, interferon regulatory factor (IRF) (which is responsible for the production of type I interferon (IFN)) (65), nuclear factor-κB (NF-κB), and the activator protein 1 signaling pathway (which is involved in the early activation of genes encoding inflammatory and endothelial cell surface molecules) are activated (66). DAMPs and PAMPs are recognized by various membrane receptors, initiating intracellular signaling cascades. PRRs encompass diverse membrane receptors, which are activated in a cell-specific manner. To identify which membrane receptor activation is responsible for initiating the intracellular signaling cascades in specific cells, we referred to Human Protein Atlas data. Among endothelial, epithelial, adaptive immune cells, and innate immune cells, innate immune cells, including Macrophages, Dendritic cells, and neutrophils, expressed TLR2, TLR4, and Dectin 1 most abundantly. endothelial protein C receptor (EPCR), Dectin 2, IL1R, and RAGE were predominantly expressed in epithelial cells. Notably, TLR2 was highly expressed in epithelial cells following innate immune cells, while TLR4 showed a sequence of expression in innate immune cells, followed by endothelial cells, and then epithelial cells. EPCR exhibited abundant expression in epithelial cells, followed by endothelial cells, adaptive immune cells, and innate immune cells, while Dectin 2 was prominently expressed in epithelial cells followed by innate immune cells. IL1R demonstrated significant expression in epithelial cells, followed by immune cells and endothelial cells, whereas RAGE showed prominent expression in epithelial cells followed by immune cells. We posit that such differential expression of these membrane receptors contributes to cell-specific membrane receptor activation. Immune cells can engage and interact with complex intracellular signaling systems, leading to the activation of the innate immune responses aimed at eliminating invading pathogens and maintaining cellular homeostasis (67). However, under the conditions in inflammatory and infectious diseases, specific host signaling pathways are drastically upregulated, resulting in the robust release of cytokines (such as tumor necrosis factor (TNF)-α, interleukin-1 (IL-1) and interlukin-6 (IL-6)) and alarmins proteins (such as high mobility group box 1 (HMGB1) and S100 family proteins) (68–70). These cytokines and alarmins act as potent mediators that contribute to immunosuppression and excessive systemic inflammation.

Numerous factors operating at the endogenous level are central to the regulation of the inflammatory response; therapeutic strategies aimed at controlling these factors are under development. Furthermore, drugs such as pentoxifylline, simvastatin, N-acetyl cysteine (NAC), tofacitinib, hydrocortisone, cobitolimod, and ulinastatin (UTI), which target endogenously positioned molecules such as phosphodiesterase, 3-hydroxy-3-methylglutaryl coenzyme A (HMG-CoA) reductase, glutathione synthetase, inhibitor of nuclear factor kappa B (IκB) kinases, Janus kinase (JAK), glucocorticoid receptor, TLR9, and serine protease, are currently the focus of clinical trials (**Table 1**) (71–77). Given the complex interplay of signaling pathways and mediators involved in inflammation, targeted therapeutic interventions are necessary to modulate these processes effectively. The following section describes how specific drugs in clinical trials aim to manipulate these mechanisms to mitigate the inflammatory response.

**Table 1:** Inflammation modulators targeting endogenous factors and clinical trials.

Compound	Target	Condition	Status	Clinical trial ID
Pentoxifylline	Phosphodiesterase	Pancreatitis	Phase 3	NCT02487225
Simvastatin	HMG-CoA reductase	Sepsis	Phase 2	NCT00528580
N-acetylcysteine	Glutathione synthetase, IκB kinases	Systemic inflammatory response syndrome	Phase 4	NCT03589495
Tofacitinib	Janus kinase	Ulcerative colitis	Phase 3	NCT01465763
Hydrocortisone	Glucocorticoid receptor	Septic shock	Phase 4	NCT02768740
Cobitolimod	Toll-like receptor 9 (TLR9)	Ulcerative colitis	Phase 3	NCT04985968
Ulinastatin	Serine protease	Septic shock	Phase 4	NCT05895240

### 4.1 Phosphodiesterase

**Pentoxifylline** - Pentoxifylline activates the adenosine receptor 2 by non-selective inhibition of phosphodiesterase enzymes and anti-inflammatory effects, leading to an increase in cyclic adenosine monophosphate (cAMP). The activation of protein kinase A (PKA) via cAMP suppresses the nuclear transfer of NF-κB and suppresses the transcription of inflammatory cytokines (78).

### 4.2 HMG-CoA reductase

**Simvastatin** - HMG-CoA reductase inhibitors (statins) may have a beneficial effect through various mechanisms of sepsis syndrome. Statin, inhibition of HMG-CoA reductase, not only reduces cholesterol levels but also decreases cholesterol-synthesis intermediates that affect intracellular signaling, cytokine expression, and chemokine regulation. This leads to reduced expression of adhesion molecules in leukocytes and endothelial cells. Additionally, statins have immune modulation and anti-coagulant effects, and evidence suggests they have a direct antimicrobial effect on bacteria (79).

### 4.3 Glutathione synthetase and IκB kinases

**N-acetyl cysteine** - NAC stimulates the synthesis of the main cell reduced Glutathione (GSH), regulates the redox state of the cell, and inhibits apoptosis caused by oxidative stress. In addition, NAC affects the NF-κB signal pathway, which targets IκB kinases (IKKα and IKKβ) that play a key role in this pathway. NAC regulation of IKKα and IKKβ is associated with the suppression of NF-κB activation induced by external stimuli such as TNF. Through these mechanisms, NAC improves the regulation of cell redox and interferes in inflammatory reactions and apoptosis processes and provides a cell protective effect (80).

### 4.4 Janus kinase

**Tofacitinib** - Tofacitinib regulates inflammation by inhibiting JAKs, which play an essential role in signaling inflammation mediators. Tofacitinib inhibits JAK1, JAK3, and to a lesser extent, JAK2. This inhibition blocks STAT protein phosphorylation, preventing cell gene expression modification. Consequently, tofacitinib regulates cytokine signaling pathways, reducing immunogenicity and inflammation in diseases like UC (81).

### 4.5 Glucocorticoid receptor

**Hydrocortisone** - Hydrocortisone binds to glucocorticoid receptors and has downstream effects such as inhibiting phospholipase A2, NF-κB, other inflammatory transcription factors, and promotion of anti-inflammatory genes. Glucocorticoids inhibit neutrophil apoptosis and decomposition, suppress phospholipase A2, reduce the formation of acoustic acid derivatives, suppress NF-κB and other inflammatory transcription factors, and promote anti-inflammatory genes such as interleukin-10 (IL-10) (82).

### 4.6 TLR9

**Cobitolimod** - Cobitolimod is a locally administered deoxyribonucleic acid (DNA)-based oligonucleotide that interacts with TLR9. Its clinical efficacy in patients with moderate-to-severe UC was demonstrated (76). Moreover, cobitolimod induces regulatory T cells in lymphocytes and antigen-presenting cells, leading to IL-10 production and the inhibition of TH-17 cells (83).

### 4.7 Serine protease

**Ulinastatin** - UTI, also known as a urinary trypsin inhibitor, inhibits serine proteases and plays a key role in reducing systemic inflammation and preventing cell apoptosis. The protease inhibitor inhibits the activation of NF-κB by reducing p38-Mitogen-activated protein kinase (MAPK) phosphorylation and promoting anti-inflammatory effects (84).

However, drugs targeting endogenous factors have the drawback of relatively slower response times (85–88). This limitation is particularly significant in the context of intracellular drug delivery, where the efficiency of drug transport across cell membranes is crucial. Furthermore, if exogenous molecules cannot pass through cell membranes, it becomes difficult to reach the cytosol (89). Unlike membrane receptors, which are located on the cell surface and interact directly with external drugs, intracellular proteins require drugs to undergo specific steps to cross the cell membrane and reach their target sites within the cell. In addition, in the c-induced sepsis animal model, the expression time of MyD88 and its downstream factors appears to be different. Experimental animal studies have shown a sequence expression of MyD88 between the cecal ligation and puncture (CLP) surgery group and the sham control group. In addition, CLP surgery significantly increased the concentration of MyD88 messenger ribonucleic acid (mRNA) in tissue in 2 hours after Sepsis induction. When MyD88-dependent TLR signaling pathways are activated, NF-κB is activated and serum cytokines, including TNF-α, are significantly increased (90). Therefore, when considering the time required to express downstream factors, drugs that block the initial signal may have a faster response time than those that affect downstream factors.

## 5 Targeting membrane receptors for rapid regulation in acute inflammatory and infectious diseases

To improve the limitations of drug response time, it is necessary to focus on the regulation of membrane receptors that initiate the inflammatory cascade. Acute inflammatory and infectious diseases are initiated by receptors located on the cellular membrane. The intracellular signaling cascades induced by membrane receptors such as PRRs drive the transcription of the inflammatory mediators that regulate the elimination of infected cells and pathogens (91). However, abnormal activation of this inflammatory system triggers septic shock or immunodeficiency (92). For example, cells rich in specific PRRs, such as TLRs, IL1R, EPCR, and others, can recognize PAMPs and DAMPs, leading to excessive inflammation that could potentially result in septic shocks. On the contrary, if endotoxin tolerance is established, even though activation is required, PRRs can remain inactive, leading to an immunosuppressive state (93, 94). Thus, the efficient regulation of membrane receptor proteins that act as PRRs might be effective as an early treatment for acute inflammatory and infectious diseases.

TLRs are the most well-known PRRs due to their crucial role in the host defense system and their involvement in various pathological processes such as sepsis (95). The activation of TLRs enables pathogen elimination through the promotion of the antibacterial activity of immune cells and the maturation of antigen-presenting cells, which involves the development of adaptive immunity (96). However, dysregulation of TLRs can lead to severe disease such as sepsis, AP, acute UC, and acute lung inflammation (27, 97, 98). TLR4 is a signal-transducing component of the lipopolysaccharide (LPS) receptor complex, which includes Myeloid differentiation factor 2 (MD-2) and cluster of differentiation 14 (CD14). Furthermore, the progression of LPS-triggered proinflammatory reactions relies solely on the endocytosis rate of TLR4 and its trafficking via the endolysosomal compartment (99). Therefore, TLR4 is considered a key therapeutic target for treating inflammatory and infectious diseases (100, 101), potentially extending to the treatment of sepsis (102). TLR2 is expressed on diverse cell surfaces, including immune cells such as macrophages, dendritic cells, and lymphocytes. The factors recognized by TLR2 include PAMPs and DAMPs (103). TLR2 is associated with the development of infectious and inflammatory diseases. TLR2 promotes the inflammatory response via activation of the MyD88/NF-κB signaling pathway (104). Furthermore, TLR2 is a therapeutic target for AP treatment (105). TLR9 was originally discovered as a sensor of bacterial DNA, which is rich in unmethylated CpG dinucleotides. However, it can also recognize the DNA released from damaged cells as a DAMPs, triggering sterile inflammation (106).

IL-1R orchestrates the signaling initiated by important IL-1 pathways involved in various pathogenic processes. Typically, IL-1R promotes the transcription of inflammatory cytokines via the canonical NF-kB pathway. However, in certain specialized cell types such as neurons, it induces inflammation independent of the NF-kB pathway (107). In addition to these membrane PRRs, EPCR negatively regulates inflammatory responses and coagulopathy in sepsis (108). Moreover, the μ-opioid receptor (MOR), which is responsible for opioid recognition, is implicated in exacerbating the immunopathology of bacterial infections (109). The triggering receptor expressed on myeloid cells-1 (TREM-1), stimulated by specific ligands such as DAMPs and PAMPs during infections or tissue damage, activates the DAP12-associated Syk signaling pathway. This activation leads to NF-κB translocation into the nucleus, which in turn upregulates the production of pro-inflammatory cytokines and chemokines. This cascade is pivotal in amplifying inflammatory responses, particularly in sepsis, where such dysregulation can exacerbate the condition (110). G-protein coupled receptors (GPCRs), a large and diverse family of transmembrane receptors, play a critical role in a wide range of physiological processes, including immune responses. These receptors are activated by various ligands, leading to conformational changes that facilitate the interaction with and activation of G-proteins. This interaction results in the dissociation of Gα and Gβγ subunits, which then modulate downstream signaling pathways, influencing cellular responses. Dysregulation of GPCR signaling has been implicated in the pathogenesis of numerous diseases, including sepsis, highlighting their potential as therapeutic targets. For example, The kinin B1 receptor is involved in sepsis-induced vascular hyperpermeability, demonstrating the nuanced roles GPCRs play in immune responses and their potential as therapeutic targets (111, 112). Also, the C5a–C5a receptor (C5aR) axis plays a critical role in inflammatory responses, especially in the context of sepsis. Upon activation by pathogens, the complement system generates C5a, a potent inflammatory peptide, which interacts with its receptor to attract immune cells like neutrophils and macrophages, leading to oxidative bursts and release of pro-inflammatory cytokines. This cascade contributes to vasodilation, tissue damage, and multiple organ failure (MOF) in acute inflammation (1, 113).

These membrane receptors are sufficient targets for drugs administered at the exogenous level and offer the potential for rapid regulatory effects owing to their high accessibility.

## 6 Membrane receptor blockade as treatment for acute inflammatory and infectious diseases

In the past, exposure to acute inflammation-related diseases primarily led to the administration of antibiotics to prevent initial infections (114). Recognizing the limitations, such as antibiotic resistance and the emergence of ‘super bugs,’ ongoing research is exploring the potential of adjunct therapies. Despite some partial effects of using biological factors in adjunct therapies, a substantial increase in survival rates was not achieved (115).. Therefore, since significant effects were not observed with biological factors alone, various experimental studies are investigating counteractive drugs targeting PAMPs and DAMPs, which are being explored for their potential to enhance the efficacy of antibiotics in treating inflammatory conditions and possibly sepsis (116–118). However, these approaches are still under investigation and have not yet been established in medical practice. Although these findings are preliminary and require further validation, the observed beneficial effects of co-administering immunomodulators with antibiotics in treating sepsis and other inflammatory infectious diseases suggest that continuing research in this direction could be promising. For example, C10-LRR shows promise as a potential new treatment option for managing overactive pro-inflammatory cytokine release by macrophages and could be effectively used to treat severe inflammatory conditions such as sepsis when combined with other therapies, including antibiotics or anti-TNFα antibodies (119). siTACE (TNF-α converting enzyme) reduces pro-inflammatory cytokines and the presence of inflammatory macrophages. TKPR-9R peptides, targeted at macrophages, enhance the cell-permeability of siRNA both *in vitro* and *in vivo*. When used together in combination therapy, siTACE/TKPR-9R complexes and antibiotics deliver simultaneous anti-inflammatory and antibacterial benefits (120). The use of a β-lactam and a macrolide together was linked to lower mortality rates in patients suffering from pneumococcal Community-acquired pneumonia (CAP) and those experiencing intense systemic inflammation. In cases where both conditions were present, the combination of β-lactam and macrolide demonstrated a protective effect against mortality in the multivariate analysis (121). Such approaches have shown potential in enhancing treatment efficacy, which supports the need for well-designed, large-scale clinical trials to confirm these benefits and establish safe and effective treatment protocols (122–125).

Acute inflammatory and infectious diseases produce a notable shift in the patterns of inflammatory mediators with disease progression, characterized by a downregulation of the expression of proinflammatory cytokines. Concurrently, DAMPs, including HMGB1, S100 family, and heat shock proteins, are upregulated (126–128). Notably, the peak TNF-α concentration occurs in the blood approximately 2 hours after endotoxin injection, whereas the concentrations of HMGB1 and S100 family protein peak around 12–18 hours after endotoxin injection (129). Moreover, in experimental studies, after TNF-α reached its peak concentration in the bloodstream, the administration of specific antibodies to inhibit HMGB1 resulted in decreased mortality rates (130).

The inhibition of the products of the inflammatory signaling pathway is effective for disease treatment. The released products, such as HMGB1, interact extracellularly with cell surface receptors such as TLRs, triggering signal transduction, which activates dendritic cells. This activation prompts the secretion of TNF-α and other proinflammatory cytokines, thereby amplifying the inflammatory response (131). Furthermore, these released products bind to the RAGE on the endothelial cell surface and induce the expression of ICAM-1 and VCAM-1, facilitating leukocyte extravasation and promoting tissue damage (132, 133). Consequently, the release of inflammatory products due to damaged endothelial or epithelial cells, along with sustained inflammation mediated by monocytes and dendritic cells, plays a critical role in the progression of sepsis toward MOF.

Taken together, these findings highlight the potential value of targeting DAMPs regulation to attenuate the detrimental effects of inflammatory responses during the later stages of acute inflammatory and infectious diseases. This suggests that in the treatment of diseases where the regulation of early inflammation is crucial, targeting the upstream inflammatory signaling pathway, rather than solely eliminating the products of the inflammatory response, may provide high-value drug candidates that overcome the challenges associated with antibiotics.

Until recently, the majority of acute inflammatory and infection diseases and DGF research had primarily centered around blocking the initial hyperinflammatory phase of conditions, which is mediated by cytokines (134–136). The following sections introduce exogenous blockers that are the most advanced membrane receptor blockers, working as early stage inflammation modulators developed for the treatment of various acute inflammatory and infectious diseases.

## 7 Membrane receptor blockade as an inflammatory regulator

### 7.1 TLR4

**Resatorvid** - A widely recognized inhibitor of TLR4 is TAK-242, which is also known as resatorvid or ethyl-(6R)-[N-(2-chloro-4-fluorophenyl) sulfamoyl], developed by Takeda Pharmaceutical Company in Osaka, Japan (137). TAK-242 interacts with cysteine 747 within the intracellular domain of TLR4. This interaction leads to the inhibition of both MyD88-dependent and MyD88-independent pathways, which are activated by LPS (138).

**Eritoran** - Eritoran acts as an antagonist of MD2-TLR4 and effectively blocks LPS-induced hyperinflammation in *in vitro* and *in vivo* experimental animal models (139). Furthermore, it reduces phosphorylated p38-MAPK and NF-κB expression levels (140).

**NI-0101** - NI-0101 forms an immune complex with the citrullinated proteins that bind to TLR4, effectively preventing cytokine release, both *in vitro* and *in vivo*. Furthermore, it successfully suppresses the anticipated increase in C-reactive protein (CRP) levels following LPS administration *in vivo* (141, 142).

### 7.2 TLR2

**OPN-305** - OPN-305 blocks TLR2/1- and TLR2/6-mediated signaling, thereby reducing the production of proinflammatory cytokines through TLR2, inducing high TLR2 occupancy, and reducing IL-6 secretion (143). In addition, its role involves the inhibition of TLR2-mediated ischemia–reperfusion injury, a pivotal factor in the pathogenesis of DGF and its subsequent complications (144).

### 7.3 IL-1R

**Anakinra** - Anakinra, an interleukin receptor antagonist, effectively inhibits the biological function of IL-1 by competitively inhibiting its binding to IL-1R (145). It is effective in diseases with severe activation of the NLRP3 inflammasome complex, as evidenced by rapid improvements in clinical symptoms and inflammatory markers, and substantial reductions in major organ-related symptoms (146).

### 7.4 EPCR

**rhAPC** - In addition to its role as an anticoagulant that downregulates thrombin generation, rhAPC binds to EPCR, activating protease-activated receptor 1 in endothelial cells, thereby inducing a multifactorial cytoprotective signaling pathway (147). Furthermore, rhAPC administration decreases the concentration of proinflammatory cytokines in the bloodstream (148).

### 7.5 MOR

**Naldemedine** - Naldemedine interacts with MOR and dose-dependently inhibits cAMP level reductions and β-arrestin recruitment increases. Moreover, naldemedine administration substantially attenuates the upregulation of genes related to immune checkpoints (149).

As mentioned in this session, compounds capable of interacting with membrane proteins under various exogenous conditions are being investigated as therapeutic agents for the treatment of acute inflammatory and infectious diseases. Furthermore, their efficacy has been substantiated in clinical trials, as described in **Table 2**.

**Table 2:** Membrane receptor blockades act as inflammation modulators and clinical trials.

Compound	Target	Condition	Status	Clinical trial ID
Eritoran	Toll-like receptor 4 (TLR4)	Sepsis	Phase 3	NCT00334828
Resatorvid		Sepsis	Phase 3	NCT00633477
NI-0101		Acute respiratory distress syndrome	Phase 2/3	NCT04401475
OPN-305	Toll-like receptor 2 (TLR2)	Delayed graft function	Phase 2	NCT01794663
Anakinra	IL-1 receptor (IL-1R)	Sepsis	Phase 2	NCT04990232
rhAPC	Endothelial protein C receptor (EPCR)	Sepsis	Phase 3	NCT00625209
Naldemedine	μ-Opioid receptor (MOR)	Pancreatitis	Phase 3	NCT04966559

Examining the outcomes of completed clinical trials, phase 3 trials revealed that eritoran was not effective in reducing mortality rates in severe sepsis patients, a finding that contradicts earlier phase 1 results and preclinical studies. TAK-242, another investigational drug, did not decrease cytokine levels or enhance organ function in patients with severe sepsis-induced shock or respiratory failure, although some mortality benefits were noted in a specific subgroup. *Post hoc* analysis of anakinra suggested a possible 30% reduction in 28-day mortality among patients with liver issues and disseminated intravascular coagulation (DIC), despite the main trial showing no overall survival benefits. Additionally, the trial involving recombinant human activated protein C indicated no advantages in treating severe septic shock. Finally, the PAMORA-RAP trial is evaluating the efficacy of naldemedine in preventing recurrent AP in a randomized controlled setup (150–154). Although initial clinical phases have shown success, there have been numerous subsequent failures. However, this does not mean that immunomodulators lack potential in treating sepsis. One possible reason for clinical failures could be that blocking the TLR4 receptor does not completely halt inflammation, as other PRRs can still recognize various PAMPs or DAMPs and activate inflammatory gene transcription independently of TLR4 signaling. Therefore, it could be beneficial to explore research modifying membrane receptor blockades to interact with a variety of PRRs, which might enable more rapid and effective control of inflammation at the start of the signaling pathway.

Although clinical trials have not yet been conducted, efforts to develop drugs targeting TREM-1 for the treatment of sepsis are underway. Peptide-based therapies such as LP17, M3, and N1 bind to TREM-1, reducing inflammatory responses and demonstrating therapeutic effects in various sepsis models induced through different methods (155–157). Various types of GPCRs are also under extensive research as targets for sepsis treatment. BI113823 is an orally active nonpeptide kinin B1 receptor antagonist of small molecule and is a potent anti-inflammatory agent with a good cardiovascular profile. BI113823 reduces systemic and tissue inflammation, prevents hemodynamic derangement, reduces multi-organ injury, and improves overall survival in the rat model of polymicrobial sepsis caused by CLP (158). The function of C5aR in sepsis was also explored using a C5aR antagonist, C5aRa. C5aRa, a cyclic peptide, competes with C5a to bind to C5aR. In cases of sepsis, C5aRa treatment hindered the chemotactic responses of neutrophils to C5a, thereby preventing C5a/C5aR-induced impairment of innate immunity, resulting in enhanced survival during a 9-day investigation. These findings additionally underscore C5aR as a prospective therapeutic target in sepsis (159, 160).

Many sepsis patients may experience a phase of excessive inflammation for a relatively brief duration. Hyperinflammation and the onset of MOF manifest over time, with early MOFs posing a risk of fatality (161). Therefore, it is crucial to promptly diagnose and initiate appropriate treatment during the early stages of sepsis. Membrane receptor blockades serve as early inflammation modulators designed for various acute inflammatory and infectious diseases. Consequently, they can swiftly counter the initial inflammatory responses in sepsis, potentially diminishing inflammation and enhancing treatment outcomes and survival rates.

## 8 Membrane receptor blockade: novel approach to address the challenge of antibiotic resistance in sepsis treatment

Conditions such as organ transplantation, UC, and AP exhibit high mortality rates due to sepsis. A swift initial intervention plays a crucial role in mitigating these mortality rates. In the context of sepsis, diagnosis and suitable management within the initial hours after onset result in notably improvements in patient outcomes (10). In the event of the onset of sepsis, antibiotic treatment must be quickly initiated, ideally within the first hour (162). Although conducting relevant culture tests before antibiotic administration is important, such diagnostic procedures should not delay antibiotic administration. The administration of an antibiotic combination effective against the presumed causative bacteria should be completed within a 3-hour timeframe (163). However, the use of antibiotic-based combination therapy is advised for a duration not exceeding 3–5 days, and transitioning to monotherapy based on the susceptibility results should be promptly considered. Generally, the antibiotic treatment period spans approximately 7–10 days (164). The constraint on antibiotic dosing regimens is that as the number of antimicrobial-resistant bacteria acquired in both community and healthcare settings increases worldwide, effective antibacterial therapy becomes increasingly difficult, especially regarding empirical antimicrobial selection (165).

In the management of acute infections and immune-related diseases, initial treatment often relies on the essential and unavoidable administration of excessive doses of antibiotics, which, over time, can create a formidable barrier known as antibiotic resistance (24, 166). Inflammatory regulators used for the treatment of acute inflammation and infectious diseases, which are applied to solve the problem of antibiotic resistance, can be categorized into drugs that regulate the entire signaling pathway and those that regulate factors at the intracellular level (167). However, in diseases such as those mentioned above where timing is crucial, medications focused on rapidly blocking signal transduction pathways may be more efficient.

Numerous obstacles exist in relying solely on antibiotic-based treatment for sepsis, which is common practice (168). Consequently, several researchers have explored the use of neutralizing-antibody-based inflammation control as adjunct therapy. However, obtaining meaningful results has been challenging owing to their diminishing efficacy over time. Furthermore, the compounds targeting endogenous factors introduced in **Table 1** have a slower response time at the *in vitro* level, making it difficult to alleviate the initial symptoms within the recommended 3-hour timeframe as stipulated by the 3-Hour Surviving Sepsis Campaign Guideline (59).

Exogenous inflammatory regulators, as described in **Table 2**, have relatively prompt inflammation control capabilities *in vitro*. In particular, regulatory substances related to TLRs, such as resatorvid and eritoran, can control inflammation within 1–2 hours of treatment (169–171). Sepsis is a frequent complication arising from combat injuries and trauma and is characterized by life-threatening organ dysfunction resulting from a dysregulated host response. Although the pathophysiology remains unclear, immunosuppression is currently acknowledged as a major cause of the high mortality rate associated with sepsis (172). From this perspective, inflammatory regulators must not permanently inhibit the inflammatory mechanism but revert to a normal state once the initial disease is alleviated. The restoration of cellular mechanisms to a normal state is evident 24 h after treatment with exogenous inflammation regulators. Additionally, the expression of TLR4, a crucial component in the recognition of secondary infectious agents following sepsis, returns to its preinhibitory state. Furthermore, the translocation of HMGB1, a significant factor in exacerbating lesions, can be inhibited at a rate ranging from 60% to 70% (30, 34, 170, 173–175). In other words, since these therapeutic candidates are not irreversible, they have the ability to modulate immunity rather than suppress it, thereby facilitating the restoration of immunity to its natural state.

In summary, patients with various acute inflammatory and infectious diseases are highly susceptible to sepsis, typically requiring the initial use of antibiotics. The frequent reliance on broad-spectrum antibiotics in high doses often leads to antibiotic resistance, a significant challenge in treatment. To address this, adjunctive therapies like immunomodulatory agents are explored, designed to be used alongside antibiotics to enhance treatment effectiveness. These agents help manage inflammation, providing valuable time to accurately identify the appropriate antibiotic treatment, thereby reducing tissue damage and mitigating antibiotic resistance. Importantly, while antibiotics target the pathogens, immunomodulatory agents control the excessive inflammatory responses that could lead to further complications. This synergistic approach allows for more precise and conservative use of antibiotics, avoiding high doses or broad-spectrum use, and protects the body from collateral damage caused by an overactive immune response. Furthermore, it is important to avoid inducing immune suppression with inflammatory regulators to prevent secondary infections and ensure the natural restoration of immune function. The novel immunomodulatory agents discussed in this paper, which target membrane receptors, present a valuable adjunct approach that enhances the efficacy of antibiotic therapy. These adjunctive therapies are not intended to replace antibiotics but to be used concurrently, enhancing their efficacy while managing inflammation effectively. It is crucial to note that in clinical trials exploring the co-administration of biological factors and antibiotics in the treatment of sepsis, antibiotics were never discontinued (176–178). Instead, these therapies are designed to complement each other, with the goal of improving outcomes by mitigating the effects of inflammation and reducing the risk of resistance development. These immunomodulatory agents, which target membrane receptors, modulate immune responses, decrease inflammation, and mitigate organ harm more rapidly than agents targeting exogenous factors. Thus, potentially reducing the need for prolonged antibiotic usage and aiding in patient recovery.

## 9 Concluding remarks

This review emphasizes the importance of early intervention in acute inflammatory and infectious diseases, particularly those associated with the risk of sepsis. AP, UC, and DGF can escalate to sepsis, resulting in high mortality rates. Timely treatment of sepsis, which is characterized by a complex interplay of proinflammatory and anti-inflammatory processes, is crucial to effectively control mortality rates. However, antibiotic resistance is a major problem with antibiotic-based therapy, which the most common sepsis treatment method. However, the efficiency of cytokine-based therapy, which was developed to address this problem, is low.

To improve these treatment methods, new methods of controlling inflammation must be developed. Current therapeutic strategies targeting endogenous molecules show promise but suffer from slow response times. To address this limitation, a novel approach focusing on membrane receptors, which are initiators of the inflammatory cascade, was proposed. Membrane receptors such as TLRs, IL-1R, EPCR, and MOR play crucial roles in the inflammatory response and provide opportunities for rapid regulation.

Various membrane receptor blockade targeting receptors such as TLR4, TLR2, IL-1R, EPCR, and MOR have shown efficacy in preclinical and clinical studies. Despite the lack of evident effects in clinical trials of TLR inhibitors for the treatment of sepsis, these failures have provided crucial information for future research. Specifically, TLR4 inhibitors such as Eritoran and Resatorvid have been safely permitted in clinical trials, laying the groundwork for subsequent studies. The failures in clinical trials have been attributed to various factors, including patient diversity, inflammation severity, and differences in infecting pathogens. Therefore, there is a suggestion for the need to explore diverse treatments and combinations in future clinical studies. And Membrane receptor blockers act as early stage inflammation modulators and produce a faster response than traditional therapies. Furthermore, the reviewed membrane-targeting substances demonstrate immunomodulatory capabilities without inducing complete immunosuppression, allowing the restoration of natural immune functioning.

**Figure 2:**
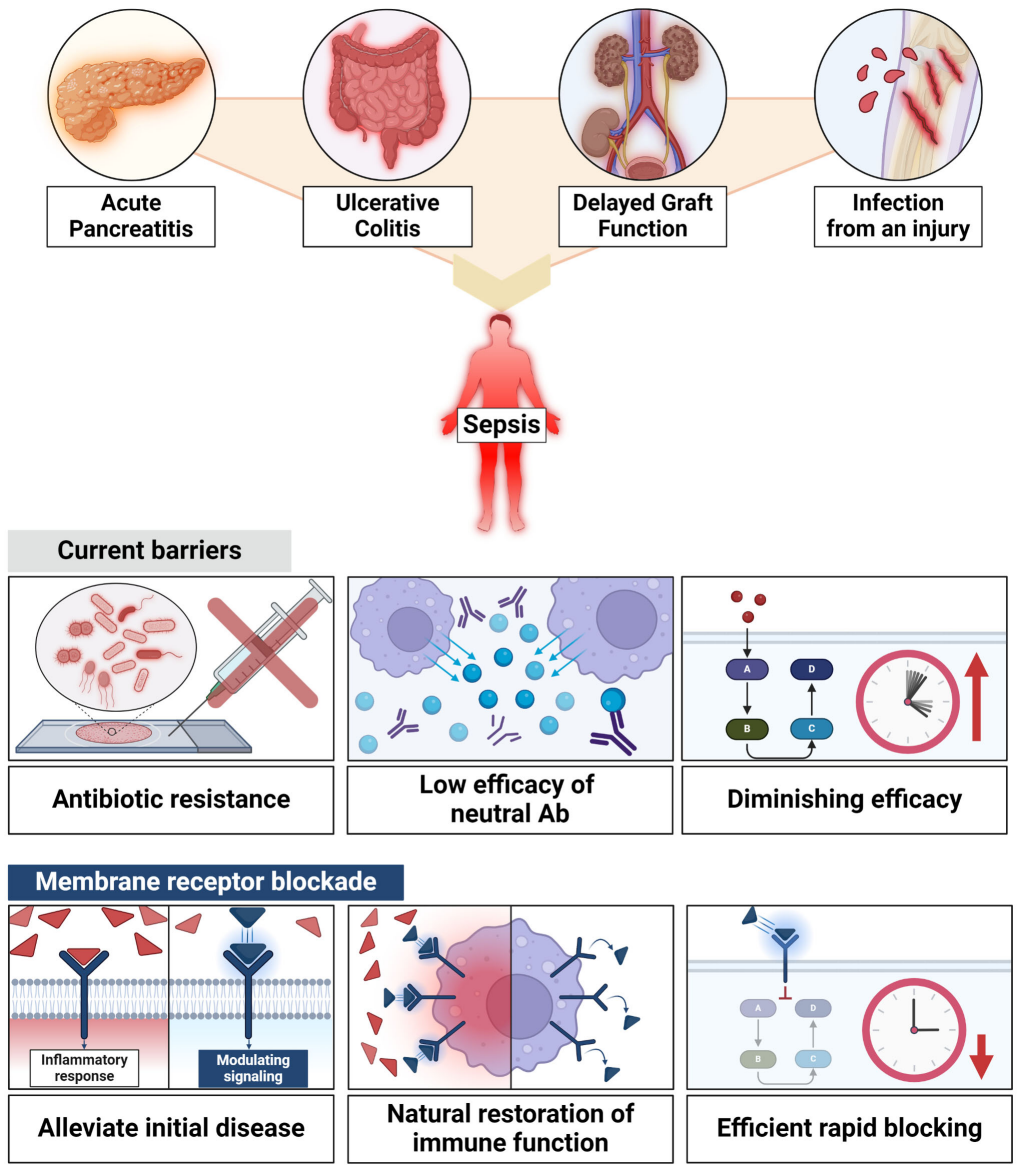
Efficacy of membrane receptor blockade. Membrane receptors blockade holds promise for enhancing patient outcomes, especially in patients prone to sepsis, all while mitigating the risks associated with antibiotic resistance and immunosuppression.

In summary, the exploration of membrane receptor blockade as an adjunct treatment for acute inflammatory and infectious diseases is a promising research avenue. When combined with antibiotics, these novel approaches have the potential to improve patient outcomes, especially in patients prone to sepsis, while minimizing the risks associated with antibiotic resistance and immunosuppression (**Figure 2**).

## Author contributions

S-JM: Conceptualization, Investigation, Writing – original draft. EC: Conceptualization, Investigation, Writing – original draft. HK: Conceptualization, Investigation, Writing – original draft. WG: Investigation, Software, Writing – original draft. C-SY: Writing – original draft, Writing – review & editing.

## References

[1] Hotchkiss RS Moldawer LL Opal SM Reinhart K Turnbull IR Vincent J-L. Sepsis and septic shock. Nat Rev Dis Primers. (2016) 2:1–21. doi: 10.1038/nrdp.2016.45 PMC5538252 28117397

[2] Gauer R Forbes D Boyer N. Sepsis: diagnosis and management. Am Family physician. (2020) 101:409–18. 32227831

[3] Wilke M Hübner C Kämmerer W. Calculated parenteral initial treatment of bacterial infections: economic aspects of antibiotic treatment. GMS Infect Dis. (2020) 8. doi: 103205/id000047 10.3205/id000047 PMC7186923 32373428

[4] Serhan CN. Treating inflammation and infection in the 21st century: new hints from decoding resolution mediators and mechanisms. FASEB J. (2017) 31:1273. doi: 10.1096/fj.201601222R 28087575 PMC5349794

[5] Gyawali B Ramakrishna K Dhamoon AS. Sepsis: The evolution in definition, pathophysiology, and management. SAGE Open Med. (2019) 7:2050312119835043. doi: 10.1177/2050312119835043 30915218 PMC6429642

[6] Bauer M Gerlach H Vogelmann T Preissing F Stiefel J Adam D. Mortality in sepsis and septic shock in Europe, North America and Australia between 2009 and 2019—results from a systematic review and meta-analysis. Crit Care. (2020) 24:1–9. doi: 10.1186/s13054-020-02950-2 32430052 PMC7236499

[7] Debela N Nekahiwot S. Sepsis, antimicrobial resistance, and alternative therapies. Am J Health Res. (2024) 12:8–18. doi: 10.11648/j.ajhr

[8] Kumar NR Balraj TA Kempegowda SN Prashant A. Multidrug-resistant sepsis: A critical healthcare challenge. Antibiotics (Basel). (2024) 13(1):46. doi: 10.3390/antibiotics13010046 38247605 PMC10812490

[9] Rudd KE Johnson SC Agesa KM Shackelford KA Tsoi D Kievlan DR. Global, regional, and national sepsis incidence and mortality, 1990-2017: analysis for the Global Burden of Disease Study. Lancet. (2020) 395:200–11. doi: 10.1016/S0140-6736(19)32989-7 PMC6970225 31954465

[10] Evans L Rhodes A Alhazzani W Antonelli M Coopersmith CM French C. Surviving sepsis campaign: international guidelines for management of sepsis and septic shock 2021. Intensive Care Med. (2021) 47:1181–247. doi: 10.1007/s00134-021-06506-y PMC8486643 34599691

[11] Gong T Liu L Jiang W Zhou R. DAMP-sensing receptors in sterile inflammation and inflammatory diseases. Nat Rev Immunol. (2020) 20:95–112. doi: 10.1038/s41577-019-0215-7 31558839

[12] Mogensen TH. Pathogen recognition and inflammatory signaling in innate immune defenses. Clin Microbiol Rev. (2009) 22:240–73. doi: 10.1128/CMR.00046-08 PMC2668232 19366914

[13] Zhang Y-Y Ning B-T. Signaling pathways and intervention therapies in sepsis. Signal Transduction Targeted Ther. (2021) 6:407. doi: 10.1038/s41392-021-00816-9 PMC8613465 34824200

[14] Chovatiya R Medzhitov R. Stress, inflammation, and defense of homeostasis. Mol Cell. (2014) 54:281–8. doi: 10.1016/j.molcel.2014.03.030 PMC4048989 24766892

[15] Karki R Kanneganti T-D. The ‘cytokine storm’: Molecular mechanisms and therapeutic prospects. Trends Immunol. (2021) 42:681–705. doi: 10.1016/j.it.2021.06.001 34217595 PMC9310545

[16] Bhat TA Panzica L Kalathil SG Thanavala Y. Immune dysfunction in patients with chronic obstructive pulmonary disease. Ann Am Thorac Soc. (2015) 12:S169–75. doi: 10.1513/AnnalsATS.201503-126AW PMC4722840 26595735

[17] Alhashem F Tiren-Verbeet NL Alp E Doganay M. Treatment of sepsis: What is the antibiotic choice in bacteremia due to carbapenem resistant Enterobacteriaceae? World J Clin cases. (2017) 5:324–32. doi: 1012998/wjccv5i8324 10.12998/wjcc.v5.i8.324 PMC5561501 28868304

[18] Alhammadi A Alshawaf R Chavda S Ramondino S Schuster M. Infectious diseases: what you may have missed in 2023. Ann Intern Med. (2024) 177:S37–s46. doi: 10.7326/M24-0679 38621246

[19] Dugar S Choudhary C Duggal A. Sepsis and septic shock: Guideline-based management. Cleve Clin J Med. (2020) 87:53–64. doi: 10.3949/ccjm.87a.18143 31990655

[20] Santacroce E D'angerio M Ciobanu AL Masini L Lo Tartaro D Coloretti I. Advances and challenges in sepsis management: modern tools and future directions. Cells. (2024) 13(5):439. doi: 10.3390/cells13050439 38474403 PMC10931424

[21] Chang CH Chang CH Huang SH Lee CS Ko PC Lin CY. Epidemiology and outcomes of multidrug-resistant bacterial infection in non-cystic fibrosis bronchiectasis. Ann Clin Microbiol Antimicrob. (2024) 23:15. doi: 10.1186/s12941-024-00675-6 38350983 PMC10865664

[22] Im Y Kang D Ko RE Lee YJ Lim SY Park S. Time-to-antibiotics and clinical outcomes in patients with sepsis and septic shock: a prospective nationwide multicenter cohort study. Crit Care. (2022) 26:19. doi: 10.1186/s13054-021-03883-0 35027073 PMC8756674

[23] Niederman MS Baron RM Bouadma L Calandra T Daneman N Dewaele J. Initial antimicrobial management of sepsis. Crit Care. (2021) 25:307. doi: 10.1186/s13054-021-03736-w 34446092 PMC8390082

[24] Legese MH Asrat D Swedberg G Hasan B Mekasha A Getahun T. Sepsis: emerging pathogens and antimicrobial resistance in Ethiopian referral hospitals. Antimicrobial Resistance Infection Control. (2022) 11:83. doi: 10.1186/s13756-022-01122-x 35698179 PMC9195281

[25] Cinel I Opal SM. Molecular biology of inflammation and sepsis: a primer. Crit Care Med. (2009) 37:291–304. doi: 10.1097/CCM.0b013e31819267fb 19050640

[26] Bosmann M Ward PA. The inflammatory response in sepsis. Trends Immunol. (2013) 34:129–36. doi: 10.1016/j.it.2012.09.004 PMC3543471 23036432

[27] El-Zayat SR Sibaii H Mannaa FA. Toll-like receptors activation, signaling, and targeting: an overview. Bull Natl Res Centre. (2019) 43:1–12. doi: 10.1186/s42269-019-0227-2

[28] Bharadwaj U Kasembeli MM Robinson P Tweardy DJ. Targeting janus kinases and signal transducer and activator of transcription 3 to treat inflammation, fibrosis, and cancer: rationale, progress, and caution. Pharmacol Rev. (2020) 72:486–526. doi: 10.1124/pr.119.018440 32198236 PMC7300325

[29] Fink MP Warren HS. Strategies to improve drug development for sepsis. Nat Rev Drug Discovery. (2014) 13:741–58. doi: 10.1038/nrd4368 25190187

[30] Samarpita S Kim JY Rasool MK Kim KS. Investigation of toll-like receptor (TLR) 4 inhibitor TAK-242 as a new potential anti-rheumatoid arthritis drug. Arthritis Res Ther. (2020) 22:1–10. doi: 10.1186/s13075-020-2097-2 31973752 PMC6979396

[31] Firmal P Shah VK Chattopadhyay S. Insight into TLR4-mediated immunomodulation in normal pregnancy and related disorders. Front Immunol. (2020) 11:807. doi: 10.3389/fimmu.2020.00807 32508811 PMC7248557

[32] Swanson L Katkar GD Tam J Pranadinata RF Chareddy Y Coates J. TLR4 signaling and macrophage inflammatory responses are dampened by GIV/Girdin. Proc Natl Acad Sci. (2020) 117:26895–906. doi: 10.1073/pnas.2011667117 PMC7604444 33055214

[33] Pan L Yu L Wang L He J Sun J Wang X. Inflammatory stimuli promote oxidative stress in pancreatic acinar cells via Toll-like receptor 4/nuclear factor-κB pathway. Int J Mol Med. (2018) 42:3582–90. doi: 10.3892/ijmm 30272284

[34] Wang Y Zhang D Li C Wu X He C Zhu X. Toll-like receptor 4-mediated endoplasmic reticulum stress induces intestinal paneth cell damage in mice following CLP-induced sepsis. Sci Rep. (2022) 12:15256. doi: 10.1038/s41598-022-19614-6 36088483 PMC9464222

[35] Wolff K Goldsmith LA Katz SI Gilchrest BA Paller AS Leffell DJ. Fitzpatrick’s dermatology in general medicine. McGraw-Hill New York: McGraw-Hill Education / Medical (2008).

[36] Li H Xie J Guo X Yang G Cai B Liu J. Bifidobacterium spp. and their metabolite lactate protect against acute pancreatitis via inhibition of pancreatic and systemic inflammatory responses. Gut Microbes. (2022) 14:2127456. doi: 10.1080/19490976.2022.2127456 36195972 PMC9542615

[37] Garg PK Singh VP. Organ failure due to systemic injury in acute pancreatitis. Gastroenterology. (2019) 156:2008–23. doi: 10.1053/j.gastro.2018.12.041 PMC6486861 30768987

[38] Flint R Windsor J. The role of the intestine in the pathophysiology and management of severe acute pancreatitis. Hpb. (2003) 5:69–85. doi: 10.1080/13651820310001108 18332961 PMC2020573

[39] Zhu M-Y Sun L-Q. Ulcerative colitis complicated with colonic necrosis, septic shock and venous thromboembolism: A case report. World J Clin cases. (2019) 7:2360. doi: 10.12998/wjcc.v7.i16.2360 31531332 PMC6718791

[40] Jahn L Schlosser M Winkler Y Foller S Grimm M-O Wolf G. Rate, factors, and outcome of delayed graft function after kidney transplantation of deceased donors. Transplant Proc. (2021) 53:1454–61. doi: 10.1016/j.transproceed.2021.01.006 33612277

[41] Ackerman KS Hoffman KL Díaz I Simmons W Ballman KV Kodiyanplakkal RP. Effect of sepsis on death as modified by solid organ transplantation. Open Forum Infect Dis. (2023) 10(4):ofad148. doi: 10.1093/ofid/ofad148 37056981 PMC10086309

[42] Tang A Shi Y Dong Q Wang S Ge Y Wang C. Prognostic differences in sepsis caused by gram-negative bacteria and gram-positive bacteria: a systematic review and meta-analysis. Crit Care. (2023) 27:467. doi: 10.1186/s13054-023-04750-w 38037118 PMC10691150

[43] Ramachandran G. Gram-positive and gram-negative bacterial toxins in sepsis: a brief review. Virulence. (2014) 5:213–8. doi: 10.4161/viru.27024 PMC3916377 24193365

[44] Hanzelmann D Joo H-S Franz-Wachtel M Hertlein T Stevanovic S Macek B. Toll-like receptor 2 activation depends on lipopeptide shedding by bacterial surfactants. Nat Commun. (2016) 7:12304. doi: 10.1038/ncomms12304 27470911 PMC4974576

[45] David MZ Daum RS. Community-associated methicillin-resistant Staphylococcus aureus: epidemiology and clinical consequences of an emerging epidemic. Clin Microbiol Rev. (2010) 23:616–87. doi: 10.1128/CMR.00081-09 PMC2901661 20610826

[46] Loof TG Goldmann O Medina E. Immune recognition of Streptococcus pyogenes by dendritic cells. Infection Immun. (2008) 76:2785–92. doi: 10.1128/IAI.01680-07 PMC2423069 18391010

[47] Chen H Zhang J He Y Lv Z Liang Z Chen J. Exploring the role of staphylococcus aureus in inflammatory diseases. Toxins. (2022) 14:464. doi: 10.3390/toxins14070464 35878202 PMC9318596

[48] Chang D Sharma L Dela Cruz CS Zhang D. Clinical epidemiology, risk factors, and control strategies of Klebsiella pneumoniae infection. Front Microbiol. (2021) 12:750662. doi: 10.3389/fmicb.2021.750662 34992583 PMC8724557

[49] Podschun R Ullmann U. Klebsiella spp. as nosocomial pathogens: epidemiology, taxonomy, typing methods, and pathogenicity factors. Clin Microbiol Rev. (1998) 11:589–603. doi: 10.1128/CMR.11.4.589 9767057 PMC88898

[50] Wei S Xu T Chen Y Zhou K. Autophagy, cell death, and cytokines in K. pneumoniae infection: therapeutic perspectives. Emerging Microbes Infections. (2023) 12:2140607. doi: 10.1080/22221751.2022.2140607 36287114 PMC9766508

[51] Shao Q Chen D Chen S Ru X Ye Q. Escherichia coli infection sepsis: an analysis of specifically expressed genes and clinical indicators. Diagnostics. (2023) 13:3542. doi: 10.3390/diagnostics13233542 38066783 PMC10706716

[52] Martin MD Skon-Hegg C Kim CY Xu J Kucaba TA Swanson W. CD115(+) monocytes protect microbially experienced mice against E. coli-induced sepsis. Cell Rep. (2023) 42:113345. doi: 101016/jcelrep2023113345 38111515 10.1016/j.celrep.2023.113345 PMC10727454

[53] Mu A Klare WP Baines SL Ignatius Pang CN Guérillot R Harbison-Price N. Integrative omics identifies conserved and pathogen-specific responses of sepsis-causing bacteria. Nat Commun. (2023) 14:1530. doi: 10.1038/s41467-023-37200-w 36934086 PMC10024524

[54] Reynolds D Kollef M. The epidemiology and pathogenesis and treatment of Pseudomonas aeruginosa infections: an update. Drugs. (2021) 81:2117–31. doi: 10.1007/s40265-021-01635-6 PMC8572145 34743315

[55] Skerrett SJ Wilson CB Liggitt HD Hajjar AM. Redundant Toll-like receptor signaling in the pulmonary host response to Pseudomonas aeruginosa. Am J Physiology-Lung Cell Mol Physiol. (2007) 292:L312–22. doi: 10.1152/ajplung.00250.2006 16936244

[56] Vincent J-L Opal SM Marshall JC Tracey KJ. Sepsis definitions: time for change. Lancet. (2013) 381:774–5. doi: 10.1016/S0140-6736(12)61815-7 PMC4535310 23472921

[57] Schuurman AR Sloot PM Wiersinga WJ Van Der Poll T. Embracing complexity in sepsis. Crit Care. (2023) 27:102. doi: 10.1186/s13054-023-04374-0 36906606 PMC10007743

[58] van der Poll T Shankar-Hari M Wiersinga WJ. The immunology of sepsis. Immunity. (2021) 54:2450–64. doi: 10.1016/j.immuni.2021.10.012 34758337

[59] Venkatesh B Schlapbach L Mason D Wilks K Seaton R Lister P. Impact of 1-hour and 3-hour sepsis time bundles on patient outcomes and antimicrobial use: A before and after cohort study. Lancet Reg Health West Pac. (2022) 18:100305. doi: 10.1016/j.lanwpc.2021.100305 35024649 PMC8654968

[60] Roh JS Sohn DH. Damage-associated molecular patterns in inflammatory diseases. Immune Netw. (2018) 18(4):e27. doi: 10.4110/in.2018.18.e27 30181915 PMC6117512

[61] Amarante-Mendes GP Adjemian S Branco LM Zanetti LC Weinlich R Bortoluci KR. Pattern recognition receptors and the host cell death molecular machinery. Front Immunol. (2018) 9:2379. doi: 10.3389/fimmu.2018.02379 30459758 PMC6232773

[62] Tobon-Velasco C Cuevas JE Torres-Ramos MA. Receptor for AGEs (RAGE) as mediator of NF-kB pathway activation in neuroinflammation and oxidative stress. CNS Neurological Disorders-Drug Targets. (2014) 13:1615–26. doi: 10.2174/1871527313666140806144831 25106630

[63] Dinarello CA. Overview of the IL-1 family in innate inflammation and acquired immunity. Immunol Rev. (2018) 281:8–27. doi: 10.1111/imr.12621 29247995 PMC5756628

[64] Ivashkiv LB Donlin LT. Regulation of type I interferon responses. Nat Rev Immunol. (2014) 14:36–49. doi: 10.1038/nri3581 24362405 PMC4084561

[65] Liu T Zhang L Joo D Sun S-C. NF-κB signaling in inflammation. Signal transduction targeted Ther. (2017) 2:1–9. doi: 10.1038/sigtrans.2017.23 PMC5661633 29158945

[66] Fujioka S Niu J Schmidt C Sclabas GM Peng B Uwagawa T. NF-κB and AP-1 connection: mechanism of NF-κB-dependent regulation of AP-1 activity. Mol Cell Biol. (2004) 24:7806–19. doi: 10.1128/MCB.24.17.7806-7819.2004 PMC507000 15314185

[67] Chaplin DD. Overview of the immune response. J Allergy Clin Immunol. (2010) 125:S3–S23. doi: 10.1016/j.jaci.2009.12.980 20176265 PMC2923430

[68] Narazaki M Kishimoto T. The two-faced cytokine IL-6 in host defense and diseases. Int J Mol Sci. (2018) 19:3528. doi: 10.3390/ijms19113528 30423923 PMC6274717

[69] Raymond SL Holden DC Mira JC Stortz JA Loftus TJ Mohr AM. Microbial recognition and danger signals in sepsis and trauma. Biochim Biophys Acta (BBA)-Molecular Basis Dis. (2017) 1863:2564–73. doi: 10.1016/j.bbadis.2017.01.013 PMC5519458 28115287

[70] Parameswaran N Patial S. Tumor necrosis factor-α signaling in macrophages. Crit Rev Eukaryot Gene Expr. (2010) 20(2):87–103. doi: 10.1615/CritRevEukarGeneExpr.v20.i2 PMC3066460 21133840

[71] Sandoval J Escobar J Pereda J Sacilotto N Rodriguez JL Sabater L. Pentoxifylline prevents loss of PP2A phosphatase activity and recruitment of histone acetyltransferases to proinflammatory genes in acute pancreatitis. J Pharmacol Exp Ther. (2009) 331:609–17. doi: 10.1124/jpet.109.157537 19671881

[72] Huang F-C Huang S-C. Differential effects of statins on inflammatory interleukin-8 and antimicrobial peptide human β-defensin 2 responses in salmonella-infected intestinal epithelial cells. Int J Mol Sci. (2018) 19:1650. doi: 10.3390/ijms19061650 29865262 PMC6032317

[73] Chao M-W Chen C-P Yang Y-H Chuang Y-C Chu T-Y Tseng C-Y. N-acetylcysteine attenuates lipopolysaccharide-induced impairment in lamination of Ctip2-and Tbr1-expressing cortical neurons in the developing rat fetal brain. Sci Rep. (2016) 6:32373. doi: 10.1038/srep32373 27577752 PMC5006028

[74] Sandborn WJ Ghosh S Panes J Vranic I Su C Rousell S. Tofacitinib, an oral Janus kinase inhibitor, in active ulcerative colitis. New Engl J Med. (2012) 367:616–24. doi: 10.1056/NEJMoa1112168 22894574

[75] Olnes MJ Kotliarov Y Biancotto A Cheung F Chen J Shi R. Effects of systemically administered hydrocortisone on the human immunome. Sci Rep. (2016) 6:1–15. doi: 10.1038/srep23002 26972611 PMC4789739

[76] Atreya R Peyrin-Biroulet L Klymenko A Augustyn M Bakulin I Slankamenac D. Cobitolimod for moderate-to-severe, left-sided ulcerative colitis (CONDUCT): a phase 2b randomised, double-blind, placebo-controlled, dose-ranging induction trial. Lancet Gastroenterol Hepatol. (2020) 5:1063–75. doi: 10.1016/S2468-1253(20)30301-0 33031757

[77] Li S-T Dai Q Zhang S-X Liu Y-J Yu Q-Q Tan F. Ulinastatin attenuates LPS-induced inflammation in mouse macrophage RAW264. 7 cells by inhibiting the JNK/NF-κB signaling pathway and activating the PI3K/Akt/Nrf2 pathway. Acta Pharmacologica Sin. (2018) 39:1294–304. doi: 10.1038/aps.2017.143 PMC6289329 29323338

[78] El−Haggar SM Hegazy SK Abd-Elsalam SM Bahaa MM. Pentoxifylline, a nonselective phosphodiesterase inhibitor, in adjunctive therapy in patients with irritable bowel syndrome treated with mebeverine. Biomedicine Pharmacotherapy. (2022) 145:112399. doi: 10.1016/j.biopha.2021.112399 34775240

[79] Pertzov B Eliakim-Raz N Atamna H Trestioreanu A Yahav D Leibovici L. Hydroxymethylglutaryl-CoA reductase inhibitors (statins) for the treatment of sepsis in adults–a systematic review and meta-analysis. Clin Microbiol Infection. (2019) 25:280–9. doi: 10.1016/j.cmi.2018.11.003 30472427

[80] Oka S-I Kamata H Kamata K Yagisawa H Hirata H. N-Acetylcysteine suppresses TNF-induced NF-κB activation through inhibition of IκB kinases. FEBS Lett. (2000) 472:196–202. doi: 10.1016/S0014-5793(00)01464-2 10788610

[81] Palasik BN Wang H. Tofacitinib, the first oral Janus kinase inhibitor approved for adult ulcerative colitis. J Pharm Pract. (2021) 34:913–21. doi: 10.1177/0897190020953019 32873116

[82] Yasir M Goyal A Sonthalia S. Corticosteroid adverse effects. Treasure Island (FL): StatPearls Publishing (2018). 30285357

[83] Zurba Y Gros B Shehab M. Exploring the pipeline of novel therapies for inflammatory bowel disease; state of the art review. Biomedicines. (2023) 11:747. doi: 10.3390/biomedicines11030747 36979724 PMC10045261

[84] Wang H Liu B Tang Y Chang P Yao L Huang B. Improvement of sepsis prognosis by ulinastatin: a systematic review and meta-analysis of randomized controlled trials. Front Pharmacol. (2019) 10:1370. doi: 10.3389/fphar.2019.01370 31849646 PMC6893897

[85] Cleary JD Evans PC Hikal AH Chapman SW. Administration of crushed extended-release pentoxifylline tablets: bioavailability and adverse effects. Am J health-system Pharm. (1999) 56:1529–34. doi: 10.1093/ajhp/56.15.1529 10478991

[86] Liu M Wang X Zhang D Yang M Han J Zhang Y. Pharmacokinetics of niacin, simvastatin and their metabolites in healthy Chinese subjects after single and multiple doses of a fixed dose combination tablet of niacin extended release/simvastatin. Drug Res. (2013) p:296–300. doi: 101055/s-0033-1357190 10.1055/s-0033-1357190 24154936

[87] Hindmarsh PC Charmandari E. Variation in absorption and half-life of hydrocortisone influence plasma cortisol concentrations. Clin Endocrinol. (2015) 82:557–61. doi: 10.1111/cen.12653 25369980

[88] Yáñez JA Remsberg CM Sayre CL Forrest ML Davies NM. Flip-flop pharmacokinetics–delivering a reversal of disposition: challenges and opportunities during drug development. Ther delivery. (2011) 2:643–72. doi: 104155/tde1119 10.4155/tde.11.19 PMC3152312 21837267

[89] Yang NJ Hinner MJ. Getting across the cell membrane: an overview for small molecules, peptides, and proteins. Site-Specific Protein Labeling: Methods Protoc. (2015) p:29–53. doi: 101007/978-1-4939-2272-7_3 10.1007/978-1-4939-2272-7_3 PMC4891184 25560066

[90] Lee E-P Lin M-J Wu H-P. Time-serial expression of toll-like receptor 4 signaling during polymicrobial sepsis in rats. Int J Immunopathology Pharmacol. (2022) 36:03946320221090021. doi: 10.1177/03946320221090021 PMC9127845 35603454

[91] Brubaker SW Bonham KS Zanoni I Kagan JC. Innate immune pattern recognition: a cell biological perspective. Annu Rev Immunol. (2015) 33:257–90. doi: 10.1146/annurev-immunol-032414-112240 PMC5146691 25581309

[92] Wei Y. Innate immunity dysregulation in myelodysplastic syndromes. Houston, TX: Defense Technical Information Center (CONTRACTING ORGANIZATION: University of Texas MD Anderson Cancer Center Houston, TX 77030, Innate Immunity Dysregulation in Myelodysplastic Syndromes by Defense Technical Information Center (2015), U.o.T.M.A.C.C.H.U. States.

[93] West MA Heagy W. Endotoxin tolerance: a review. Crit Care Med. (2002) 30:S64–73. doi: 10.1097/00003246-200201001-00009 11782563

[94] Seibl R Kyburz D Lauener RP Gay S. Pattern recognition receptors and their involvement in the pathogenesis of arthritis. Curr Opin Rheumatol. (2004) 16:411–8. doi: 10.1097/01.bor.0000127108.08398.34 15201605

[95] Li D Wu M. Pattern recognition receptors in health and diseases. Signal transduction targeted Ther. (2021) 6:291. doi: 10.1038/s41392-021-00687-0 PMC8333067 34344870

[96] MG N. Toll-like receptors and the host defense against microbial pathogens: bringing specificity to the innate-immune system. J Leukoc Biol. (2004) 75:749–55. doi: 10.1189/jlb.1103543 15075354

[97] Venkatesh K Glenn H Delaney A Andersen CR Sasson SC. Fire in the belly: A scoping review of the immunopathological mechanisms of acute pancreatitis. Front Immunol. (2023) 13:1077414. doi: 10.3389/fimmu.2022.1077414 36713404 PMC9874226

[98] Lafferty EI Qureshi ST Schnare M. The role of toll-like receptors in acute and chronic lung inflammation. J Inflammation. (2010) 7:1–14. doi: 10.1186/1476-9255-7-57 PMC3003652 21108806

[99] Ciesielska A Matyjek M Kwiatkowska K. TLR4 and CD14 trafficking and its influence on LPS-induced pro-inflammatory signaling. Cell Mol Life Sci. (2021) 78:1233–61. doi: 10.1007/s00018-020-03656-y PMC7904555 33057840

[100] Wittebole X Castanares-Zapatero D Laterre P-F. Toll-like receptor 4 modulation as a strategy to treat sepsis. Mediators Inflamm. (2010) 2010:9. doi: 10.1155/2010/568396 PMC2855078 20396414

[101] Bai Y Min R Chen P Mei S Deng F Zheng Z. Disulfiram blocks inflammatory TLR4 signaling by targeting MD-2. Proc Natl Acad Sci. (2023) 120:e2306399120. doi: 10.1073/pnas.2306399120 37487070 PMC10401014

[102] Kuzmich NN Sivak KV Chubarev VN Porozov YB Savateeva-Lyubimova TN Peri F. TLR4 signaling pathway modulators as potential therapeutics in inflammation and sepsis. Vaccines. (2017) 5:34. doi: 10.3390/vaccines5040034 28976923 PMC5748601

[103] Oliveira-Nascimento L Massari P Wetzler LM. The role of TLR2 in infection and immunity. Front Immunol. (2012) 3:79. doi: 10.3389/fimmu.2012.00079 22566960 PMC3342043

[104] Kuriakose S Onyilagha C Singh R Olayinka-Adefemi F Jia P Uzonna JE. TLR-2 and MyD88-dependent activation of MAPK and STAT proteins regulates proinflammatory cytokine response and immunity to experimental trypanosoma congolense infection. Front Immunol. (2019) 10:2673. doi: 10.3389/fimmu.2019.02673 31824484 PMC6883972

[105] Li L Liu Q Le C Zhang H Liu W Gu Y. Toll-like receptor 2 deficiency alleviates acute pancreatitis by inactivating the NF-κB/NLRP3 pathway. Int Immunopharmacol. (2023) 121:110547. doi: 10.1016/j.intimp.2023.110547 37356124

[106] Shintani Y Kapoor A Kaneko M Smolenski RT D’acquisto F Coppen SR. TLR9 mediates cellular protection by modulating energy metabolism in cardiomyocytes and neurons. Proc Natl Acad Sci. (2013) 110:5109–14. doi: 10.1073/pnas.1219243110 PMC3612600 23479602

[107] Liu X Nemeth DP Mckim DB Zhu L Disabato DJ Berdysz O. Cell-type-specific interleukin 1 receptor 1 signaling in the brain regulates distinct neuroimmune activities. Immunity. (2019) 50:317–333. e6. doi: 10.1016/j.immuni.2018.12.012 30683620 PMC6759085

[108] Guitton C Gérard N Sébille V Bretonnière C Zambon O Villers D. Early rise in circulating endothelial protein C receptor correlates with poor outcome in severe sepsis. Intensive Care Med. (2011) 37:950–6. doi: 10.1007/s00134-011-2171-y PMC3529933 21394629

[109] Chang SL Beltran JA Swarup S. Expression of the mu opioid receptor in the human immunodeficiency virus type 1 transgenic rat model. J Virol. (2007) 81:8406–11. doi: 10.1128/JVI.00155-07 PMC1951376 17553897

[110] Siskind S Brenner M Wang P. TREM-1 modulation strategies for sepsis. Front Immunol. (2022) 13:907387. doi: 10.3389/fimmu.2022.907387 35784361 PMC9240770

[111] Rehman A Baloch NU-A Morrow JP Pacher P Haskó G. Targeting of G-protein coupled receptors in sepsis. Pharmacol Ther. (2020) 211:107529. doi: 10.1016/j.pharmthera.2020.107529 32197794 PMC7388546

[112] Ruiz S Vardon-Bounes F Buléon M Guilbeau-Frugier C Séguelas M-H Conil J-M. Kinin B1 receptor: a potential therapeutic target in sepsis-induced vascular hyperpermeability. J Trans Med. (2020) 18:1–16. doi: 10.1186/s12967-020-02342-8 PMC7168845 32306971

[113] Sommerfeld O Medyukhina A Neugebauer S Ghait M Ulferts S Lupp A. Targeting complement C5a receptor 1 for the treatment of immunosuppression in sepsis. Mol Ther. (2021) 29:338–46. doi: 10.1016/j.ymthe.2020.09.008 PMC7791006 32966769

[114] Gregoriano C Heilmann E Molitor A Schuetz P. Role of procalcitonin use in the management of sepsis. J Thorac Dis. (2020) 12:S5. doi: 10.21037/jtd 32148921 PMC7024752

[115] Yoon JY Kwon JY. Inflammation and sepsis. Acute Crit Care. (2010) 25:1–8. doi: 10.4266/kjccm.2010.25.1.1

[116] Lv S Han M Yi R Kwon S Dai C Wang R. Anti-TNF-α therapy for patients with sepsis: a systematic meta-analysis. Int J Clin Pract. (2014) 68:520–8. doi: 10.1111/ijcp.12382 24548627

[117] Lu R-M Hwang Y-C Liu I-J Lee C-C Tsai H-Z Li H-J. Development of therapeutic antibodies for the treatment of diseases. J Biomed Sci. (2020) 27:1–30. doi: 10.1186/s12929-019-0592-z 31894001 PMC6939334

[118] Aslam B Wang W Arshad MI Khurshid M Muzammil S Rasool MH. Antibiotic resistance: a rundown of a global crisis. Infect Drug Resist. (2018) 11:1645–58. doi: 10.2147/IDR PMC6188119 30349322

[119] Koo JH Kim SH Jeon SH Kang MJ Choi JM. Macrophage-preferable delivery of the leucine-rich repeat domain of NLRX1 ameliorates lethal sepsis by regulating NF-κB and inflammasome signaling activation. Biomaterials. (2021) 274:120845. doi: 10.1016/j.biomaterials.2021.120845 33971559

[120] Lee J Son W Hong J Song Y Yang CS Kim YH. Down-regulation of TNF-α via macrophage-targeted RNAi system for the treatment of acute inflammatory sepsis. J Control Release. (2021) 336:344–53. doi: 10.1016/j.jconrel.2021.06.022 34147573

[121] Ceccato A Cilloniz C Martin-Loeches I Ranzani OT Gabarrus A Bueno L. Effect of combined β-lactam/macrolide therapy on mortality according to the microbial etiology and inflammatory status of patients with community-acquired pneumonia. Chest. (2019) 155:795–804. doi: 10.1016/j.chest.2018.11.006 30471269

[122] Soni M Handa M Singh KK Shukla R. Recent nanoengineered diagnostic and therapeutic advancements in management of Sepsis. J Control Release. (2022) 352:931–45. doi: 10.1016/j.jconrel.2022.10.029 PMC9665001 36273527

[123] Garzón-Tituaña M Sierra-Monzón JL Comas L Santiago L Khaliulina-Ushakova T Uranga-Murillo I. Granzyme A inhibition reduces inflammation and increases survival during abdominal sepsis. Theranostics. (2021) 11:3781–95. doi: 10.7150/thno.49288 PMC7914344 33664861

[124] Lima CX Souza DG Amaral FA Fagundes CT Rodrigues IP Alves-Filho JC. Therapeutic effects of treatment with anti-TLR2 and anti-TLR4 monoclonal antibodies in polymicrobial sepsis. PloS One. (2015) 10:e0132336. doi: 10.1371/journal.pone.0132336 26147469 PMC4492955

[125] Pant A Mackraj I Govender T. Advances in sepsis diagnosis and management: a paradigm shift towards nanotechnology. J BioMed Sci. (2021) 28:6. doi: 10.1186/s12929-020-00702-6 33413364 PMC7790597

[126] Karakike E Adami M-E Lada M Gkavogianni T Koutelidakis IM Bauer M. Late peaks of HMGB1 and sepsis outcome: evidence for synergy with chronic inflammatory disorders. Shock. (2019) 52:334–9. doi: 10.1097/SHK.0000000000001265 30239421

[127] Wu L Feng Q Ai M-L Deng S-Y Liu Z-Y Huang L. The dynamic change of serum S100B levels from day 1 to day 3 is more associated with sepsis-associated encephalopathy. Sci Rep. (2020) 10:7718. doi: 10.1038/s41598-020-64200-3 32382007 PMC7206038

[128] Fitrolaki M-D Dimitriou H Venihaki M Katrinaki M Ilia S Briassoulis G. Increased extracellular heat shock protein 90α in severe sepsis and SIRS associated with multiple organ failure and related to acute inflammatory-metabolic stress response in children. Medicine. (2016) 95:35. doi: 10.1097/MD.0000000000004651 PMC5008570 27583886

[129] Borovikova LV Ivanova S Zhang M Yang H Botchkina GI Watkins LR. Vagus nerve stimulation attenuates the systemic inflammatory response to endotoxin. Nature. (2000) 405:458–62. doi: 10.1038/35013070 10839541

[130] Wang H Bloom O Zhang M Vishnubhakat JM Ombrellino M Che J. HMG-1 as a late mediator of endotoxin lethality in mice. Science. (1999) 285:248–51. doi: 10.1126/science.285.5425.248 10398600

[131] Andersson U Tracey KJ. HMGB1 is a therapeutic target for sterile inflammation and infection. Annu Rev Immunol. (2011) 29:139–62. doi: 10.1146/annurev-immunol-030409-101323 PMC4536551 21219181

[132] Stanzione R Forte M Cotugno M Bianchi F Marchitti S Rubattu S. Role of DAMPs and of leukocytes infiltration in ischemic stroke: insights from animal models and translation to the human disease. Cell Mol Neurobiol. (2022) 42:545–56. doi: 10.1007/s10571-020-00966-4 PMC11441194 32996044

[133] Kim Y Cho AY Kim HC Ryu D Jo SA Jung Y-S. Effects of natural polyphenols on oxidative stress-mediated blood-brain barrier dysfunction. Antioxidants. (2022) 11:197. doi: 10.3390/antiox11020197 35204080 PMC8868362

[134] Hotchkiss RS Opal SM. Immunotherapy for sepsis: a new approach against an ancient foe. New Engl J Med. (2010) 363:87. doi: 10.1056/NEJMcibr1004371 20592301 PMC4136660

[135] Payen D Monneret G Hotchkiss R. Immunotherapy-a potential new way forward in the treatment of sepsis. Crit Care. (2013) 17:1–2. doi: 10.1186/cc12490 PMC4056021 23425441

[136] Ferdinand JR Hosgood SA Moore T Ferro A Ward CJ Castro‐Dopico T. Cytokine absorption during human kidney perfusion reduces delayed graft function–associated inflammatory gene signature. Am J Transplant. (2021) 21:2188–99. doi: 10.1111/ajt.16371 PMC8246774 33098231

[137] Savva A Roger T. Targeting toll-like receptors: promising therapeutic strategies for the management of sepsis-associated pathology and infectious diseases. Front Immunol. (2013) 4:387. doi: 10.3389/fimmu.2013.00387 24302927 PMC3831162

[138] Bayan N Yazdanpanah N Rezaei N. Role of toll-like receptor 4 in diabetic retinopathy. Pharmacol Res. (2022) 175:105960. doi: 10.1016/j.phrs.2021.105960 34718133

[139] Barochia A Solomon S Cui X Natanson C Eichacker PQ. Eritoran tetrasodium (E5564) treatment for sepsis: review of preclinical and clinical studies. Expert Opin Drug Metab Toxicol. (2011) 7:479–94. doi: 10.1517/17425255.2011.558190 PMC3065179 21323610

[140] Yousif N Hadi N Al-Amran F Zigam Q. Cardioprotective effects of irbesartan in polymicrobial sepsis. Herz. (2018) 43:140–5. doi: 10.1007/s00059-017-4537-6 28144715

[141] Monnet E Lapeyre G Poelgeest EV Jacqmin P Graaf KD Reijers J. Evidence of NI-0101 pharmacological activity, an anti-TLR4 antibody, in a randomized phase I dose escalation study in healthy volunteers receiving LPS. Clin Pharmacol Ther. (2017) 101:200–8. doi: 10.1002/cpt.522 27706798

[142] Monnet E Choy EH Mcinnes I Kobakhidze T De Graaf K Jacqmin P. Efficacy and safety of NI-0101, an anti-toll-like receptor 4 monoclonal antibody, in patients with rheumatoid arthritis after inadequate response to methotrexate: a phase II study. Ann rheumatic Dis. (2020) 79:316–23. doi: 10.1136/annrheumdis-2019-216487 31892533

[143] Bansal A Mostafa MM Kooi C Sasse SK Michi AN Shah SV. Interplay between nuclear factor-κB, p38 MAPK, and glucocorticoid receptor signaling synergistically induces functional TLR2 in lung epithelial cells. J Biol Chem. (2022) 298(4):101747. doi: 10.1016/j.jbc.2022.101747 35189144 PMC8942839

[144] Salvadori M Rosso G Bertoni E. Update on ischemia-reperfusion injury in kidney transplantation: Pathogenesis and treatment. World J Transplant. (2015) 5:52. doi: 10.5500/wjt.v5.i2.52 26131407 PMC4478600

[145] Kaneko N Kurata M Yamamoto T Morikawa S Masumoto J. The role of interleukin-1 in general pathology. Inflammation regeneration. (2019) 39:1–16. doi: 10.1186/s41232-019-0101-5 31182982 PMC6551897

[146] Bachove I Chang C. Anakinra and related drugs targeting interleukin-1 in the treatment of cryopyrin-associated periodic syndromes. Open Access Rheumatology: Res Rev. (2014) p:15–25. doi: 102147/OARRRS46017 10.2147/OARRR.S46017 PMC5045113 27790031

[147] Giri H Biswas I Rezaie AR. Activated protein C inhibits mesothelial-to-mesenchymal transition in experimental peritoneal fibrosis. J Thromb Haemostasis. (2023) 21:133–44. doi: 10.1016/j.jtha.2022.10.012 PMC10726528 36695376

[148] Boissier M-C Semerano L. From coagulation to inflammation: novel avenues for treating rheumatoid arthritis with activated protein C. Rheumatology. (2019) 58(10):1710–1. doi: 10.1093/rheumatology/kez200 31121036

[149] Gondoh E Hamada Y Mori T Iwazawa Y Shinohara A Narita M. Possible mechanism for improving the endogenous immune system through the blockade of peripheral μ-opioid receptors by treatment with naldemedine. Br J Cancer. (2022) 127:1565–74. doi: 10.1038/s41416-022-01928-x PMC9553910 35945243

[150] Opal SM Laterre PF Francois B Larosa SP Angus DC Mira JP. Effect of eritoran, an antagonist of MD2-TLR4, on mortality in patients with severe sepsis: the ACCESS randomized trial. Jama. (2013) 309:1154–62. doi: 10.1001/jama.2013.2194 23512062

[151] Rice TW Wheeler AP Bernard GR Vincent JL Angus DC Aikawa N. A randomized, double-blind, placebo-controlled trial of TAK-242 for the treatment of severe sepsis. Crit Care Med. (2010) 38:1685–94. doi: 10.1097/CCM.0b013e3181e7c5c9 20562702

[152] Leventogiannis K Kyriazopoulou E Antonakos N Kotsaki A Tsangaris I Markopoulou D. Toward personalized immunotherapy in sepsis: The PROVIDE randomized clinical trial. Cell Rep Med. (2022) 3:100817. doi: 10.1016/j.xcrm.2022.100817 36384100 PMC9729870

[153] Annane D Timsit JF Megarbane B Martin C Misset B Mourvillier B. Recombinant human activated protein C for adults with septic shock: a randomized controlled trial. Am J Respir Crit Care Med. (2013) 187:1091–7. doi: 10.1164/rccm.201211-2020OC 23525934

[154] Cook ME Knoph CS Fjelsted CA Frøkjær JB Bilgrau AE Novovic S. Effects of a peripherally acting µ-opioid receptor antagonist for the prevention of recurrent acute pancreatitis: study protocol for an investigator-initiated, randomized, placebo-controlled, double-blind clinical trial (PAMORA-RAP trial). Trials. (2023) 24:301. doi: 10.1186/s13063-023-07287-z 37127657 PMC10150502

[155] Gibot S Kolopp-Sarda M-N Béné M-C Bollaert P-E Lozniewski A Mory F. A soluble form of the triggering receptor expressed on myeloid cells-1 modulates the inflammatory response in murine sepsis. J Exp Med. (2004) 200:1419–26. doi: 10.1084/jem.20040708 PMC2211948 15557347

[156] Denning N-L Aziz M Murao A Gurien SD Ochani M Prince JM. Extracellular CIRP as an endogenous TREM-1 ligand to fuel inflammation in sepsis. JCI Insight. (2020) 5(5):e134172. doi: 10.1172/jci.insight.134172 32027618 PMC7141396

[157] Sharapova TN Romanova EA Chernov AS Minakov AN Kazakov VA Kudriaeva AA. Protein PGLYRP1/Tag7 peptides decrease the proinflammatory response in human blood cells and mouse model of diffuse alveolar damage of lung through blockage of the TREM-1 and TNFR1 receptors. Int J Mol Sci. (2021) 22:11213. doi: 10.3390/ijms222011213 34681871 PMC8538247

[158] Murugesan P Jung B Lee D Khang G Doods H Wu D. Kinin B1 receptor inhibition with BI113823 reduces inflammatory response, mitigates organ injury, and improves survival among rats with severe sepsis. J Infect Dis. (2016) 213:532–40. doi: 10.1093/infdis/jiv426 26310310

[159] Yan C Gao H. New insights for C5a and C5a receptors in sepsis. Front Immunol. (2012) 3:368. doi: 10.3389/fimmu.2012.00368 23233853 PMC3518060

[160] Huber-Lang MS Riedeman NC Sarma JV Younkin EM Mcguire SR Laudes IJ. Protection of innate immunity by C5aR antagonist in septic mice. FASEB J. (2002) 16(12):1477–694. doi: 10.1096/fj.02-0209com 12374779

[161] Cao M Wang G Xie J. Immune dysregulation in sepsis: experiences, lessons and perspectives. Cell Death Discovery. (2023) 9:465. doi: 10.1038/s41420-023-01766-7 38114466 PMC10730904

[162] Ammar MA Ammar AA Wieruszewski PM Bissell BD Long T M Albert L. Timing of vasoactive agents and corticosteroid initiation in septic shock. Ann Intensive Care. (2022) 12:47. doi: 10.1186/s13613-022-01021-9 35644899 PMC9148864

[163] Seymour CW Gesten F Prescott HC Friedrich ME Iwashyna TJ Phillips GS. Time to treatment and mortality during mandated emergency care for sepsis. New Engl J Med. (2017) 376:2235–44. doi: 10.1056/NEJMoa1703058 PMC5538258 28528569

[164] Park S Hong S-B. Treatment guidelines of severe sepsis and septic shock. J Neurocritical Care. (2015) 8:9–15. doi: 10.18700/jnc.2015.8.1.9

[165] Pradipta IS Sodik DC Lestari K Parwati I Halimah E Diantini A. Antibiotic resistance in sepsis patients: evaluation and recommendation of antibiotic use. North Am J Med Sci. (2013) 5:344. doi: 10.4103/1947-2714.114165 PMC3731864 23923107

[166] Rossiter SE Fletcher MH Wuest WM. Natural products as platforms to overcome antibiotic resistance. Chem Rev. (2017) 117:12415–74. doi: 10.1021/acs.chemrev.7b00283 PMC5869711 28953368

[167] Stanke-Labesque F Gautier-Veyret E Chhun S Guilhaumou R Pharmacology, FSO, Therapeutics. Inflammation is a major regulator of drug metabolizing enzymes and transporters: consequences for the personalization of drug treatment. Pharmacol Ther. (2020) 215:107627. doi: 10.1016/j.pharmthera.2020.107627 32659304 PMC7351663

[168] Martínez ML Plata-Menchaca EP Ruiz-Rodríguez JC Ferrer R. An approach to antibiotic treatment in patients with sepsis. J Thorac Dis. (2020) 12:1007. doi: 10.21037/jtd 32274170 PMC7139065

[169] Ono Y Maejima Y Saito M Sakamoto K Horita S Shimomura K. TAK-242, a specific inhibitor of Toll-like receptor 4 signalling, prevents endotoxemia-induced skeletal muscle wasting in mice. Sci Rep. (2020) 10:694. doi: 10.1038/s41598-020-57714-3 31959927 PMC6970997

[170] McDonald K-A Huang H Tohme S Loughran P Ferrero K Billiar T. Toll-like receptor 4 (TLR4) antagonist eritoran tetrasodium attenuates liver ischemia and reperfusion injury through inhibition of high-mobility group box protein B1 (HMGB1) signaling. Mol Med. (2014) 20:639–48. doi: 10.2119/molmed.2014.00076 PMC4365061 25375408

[171] Matsunaga N Tsuchimori N Matsumoto T Ii M. TAK-242 (resatorvid), a small-molecule inhibitor of Toll-like receptor (TLR) 4 signaling, binds selectively to TLR4 and interferes with interactions between TLR4 and its adaptor molecules. Mol Pharmacol. (2011) 79:34–41. doi: 10.1124/mol.110.068064 20881006

[172] Liu D Huang S-Y Sun J-H Zhang H-C Cai Q-L Gao C. Sepsis-induced immunosuppression: mechanisms, diagnosis and current treatment options. Military Med Res. (2022) 9:1–19. doi: 10.1186/s40779-022-00422-y PMC9547753 36209190

[173] Xing Z Zhen T Jie F Jie Y Shiqi L Kaiyi Z. Early Toll-like receptor 4 inhibition improves immune dysfunction in the hippocampus after hypoxic-ischemic brain damage. Int J Med Sci. (2022) 19:142. doi: 10.7150/ijms.66494 34975308 PMC8692118

[174] Chang C Hu L Sun S Song Y Liu S Wang J. Regulatory role of the TLR4/JNK signaling pathway in sepsis−induced myocardial dysfunction. Mol Med Rep. (2021) 23:1–10. doi: 10.3892/mmr PMC7974310 33760172

[175] Hsieh Y-C Lee K-C Wu P-S Huo T-I Huang Y-H Hou M-C. Eritoran attenuates hepatic inflammation and fibrosis in mice with chronic liver injury. Cells. (2021) 10:1562. doi: 10.3390/cells10061562 34205789 PMC8235164

[176] Richardson PG Perrot A San-Miguel J Beksac M Spicka I Leleu X. Isatuximab plus pomalidomide and low-dose dexamethasone versus pomalidomide and low-dose dexamethasone in patients with relapsed and refractory multiple myeloma (ICARIA-MM): follow-up analysis of a randomised, phase 3 study. Lancet Oncol. (2022) 23:416–27. doi: 10.1016/S1470-2045(22)00019-5 35151415

[177] Sehgal IS Basumatary NM Dhooria S Prasad KT Muthu V Aggarwal AN. A randomized trial of mycobacterium w in severe presumed gram-negative sepsis. Chest. (2021) 160:1282–91. doi: 10.1016/j.chest.2021.03.062 33852919

[178] Annane D Renault A Brun-Buisson C Megarbane B Quenot JP Siami S. Hydrocortisone plus fludrocortisone for adults with septic shock. N Engl J Med. (2018) 378:809–18. doi: 10.1056/NEJMoa1705716 29490185

